# Tear secretion dysfunction among women workers engaged in light-on tests in the TFT-LCD industry

**DOI:** 10.1186/1471-2458-6-303

**Published:** 2006-12-16

**Authors:** Shih-Bin Su, Chih-Wei Lu, Jiunn-Woei Sheen, Shu-Chun Kuo, How-Ran Guo

**Affiliations:** 1 Department of Environmental and Occupational Health, Medical College, National Cheng Kung University, 138 Sheng-Li Road, Tainan, Taiwan; 2 Tainan Science Industrial Park Clinic, Chi-Mei Medical Center, Tainan, Taiwan; 3 Graduate Institute of Occupational Safety and Health, Kaohsiung Medical University, Kaohsiung, Taiwan; 4 Department of Ophthalmology, Chi-Mei Medical Center, Tainan, Taiwan

## Abstract

**Background:**

The TFT-LCD (thin film transistor liquid crystal display) industry is rapidly growing in Taiwan and many other countries. A large number of workers, mainly women, are employed in the light-on test process to detect the defects of products. At the light-on test workstation, the operator is generally exposed to low humidity (in the clean room environment), flashing light, and low ambient illumination for long working hours. Many workers complained about eye discomfort, and therefore we conducted a study to evaluate the tear secretion function of light-on test workers of a TFT-LCD company.

**Methods:**

We recruited workers engaged in light-on tests in the company during their periodical health examination. In addition to a questionnaire survey of demographic characteristics and ophthalmic symptoms, we evaluated the tear secretion function of both eyes of each participant using the Schirmer's lacrimal basal secretion test with anaesthesia. A participant with one or both eyes yielding abnormal test results was defined as a case of tear secretion dysfunction.

**Results:**

During the study period, a total of 371 light-on test workers received the health examination at the clinic of the park, and 52 of them were excluded due to having ophthalmic diseases and other systemic diseases that may affect ophthalmic function. All the remaining 319 qualified workers agreed to participate in this study, and they were all females working by 4-shift rotations. The average age was 24.2 years old (standard deviation [SD] = 3.8), and the average employment duration was 13.6 months (SD = 5.7). Among the 11 ophthalmic symptoms evaluated, eye dryness was the most prevalent (prevalence = 43.3%). In addition, the prevalence of tear secretion dysfunction in at least one eye was 40.1% (128 cases), and contact lens users had an odds ratio of 1.73 (95% confidence interval = 1.02–2.94) in comparison with non-contact lens users. Comparing the Schirmer's test results of those who also participated in the screening in the previous year, we found 40 of the 156 participants (17.2%) with normal test results in the previous year turned abnormal in 2001. In contrast, only 21 of the 76 participants (9.1%) with abnormal test results in the previous year turned normal, and the difference was statistically significant (p = 0.02 for McNemar's test).

**Conclusion:**

The prevalence of tear secretion dysfunction in woman workers engaged in light-on tests is high and increases with a one-year duration of employment. The use of contact lens may further increase the risk.

## Background

TFT-LCD (thin film transistor-liquid crystal display) industry has been rapidly growing in Taiwan and many countries in the world. Taiwan's global market share of LCD panels has reached 35.4% in 2003, which made Taiwan the second leading panel producer, next only to Korea [1]. The manufacturing of TFT-LCD involves array process (array), panel process (cell) and module assembly process (module). To ensure a high yield rate of products and reduce the costs, the manufacturers exercise strict quality assurance and quality control measures, and the careful inspection of panels plays a very important role in the whole manufacturing process. Panels with defects or imperfects can be picked up through strict inspections and then repaired or recycled.

Methods of inspecting the panels include optics instrumental examination, electric instrumental examination, and human eye check [2-4]. The eye check (light-on test) is to detect panel's defects by human eyes. In general, it includes the gross inspection of the panel first, which is followed by inspection of the surface of panel, modules, and the panel's picture display quality by microscopy.

The light-on test is one of the most important quality control steps during the panel process and module assembly in the manufacturing of TFT-LCD. In this test, the inspector controls the power switch of the light source of the product and, under the maximum electric current, lets the board illuminate red, green and blue for color testing and white, dark, and gray for monochrome testing. Because some minor defects only appear under extreme operation conditions, such a procedure is required to ensure a high quality of the product. It is called "light-on" test because the test involves turning on the light source of the product, in contrast with others that do not. The test is performed by human eyes, and workers, mostly women, generally carry out the task in a dark environment, which may cause eye strain and even lead to poor eyesight, throughout the shift with limited resting time [5,6]. Furthermore, to ensure high quality and high yield, the TFT-LCD industry places most manufacturing processes in so-called "clean rooms." In clean rooms, the dust particles in the air are controlled to extremely low levels, and the temperature and the humidity are maintained at relatively lower levels in comparison with the general ambient environment in Taiwan. In addition, to prevent the contamination of dusts, clean room workers have to put on special clothing covering the whole body from head to toes [5].

Many workers complained about eye discomforts such as dryness and soreness, but the ophthalmic effects of working in such strictly controlled environments are not clear. To evaluate whether the clean room workers performing light-on tests have a high risk of developing tear secretion dysfunction and eye symptoms, we conducted a study in a TFT-LCD company in Taiwan. This study was in fact based on a work-place sponsored eye-health event, in which the employer and employees agree to include the evaluation of tear secretion function in the routine annual health check-up.

## Methods

We recruited workers of a TFT-LCD company located in the Tainan Science Industrial Park who received the annual routine health examination between June 1 and August 31, 2001. In addition to the medical history taken by the physician as a regular component of the health check-up, participants were asked to fill out a standard questionnaire, which collected information on demographic characteristics and ophthalmic symptoms, including eye dryness, eye itching, red eyes, eye pain, blurred vision at far distance, increased discharge, floaters, double vision, unable to focus from near to far, change of color perception, and blurred vision at near distance. Participants with history of ophthalmic diseases (including presbyopia, high myopia above 8 diopers, strabismus, amblyopia, glaucoma, color blind, and achromatopsia) and other systemic diseases that may affect ophthalmic function were excluded. According to the process in which the light-on tests were performed, the participants were divided into LCD station workers (in the panel process) and LCM station workers (in the module assembly). While these two types of stations have similar humidity (relative humidity 55 ± 5%) and workers perform the same task, LCD stations have lower environment illumination (4.0 vs. 129 Lux).

The health examination also included physical examination by experienced ophthalmologist, eyesight check, intraocular pressure measurement, and optic fundus inspection. The Schirmer's tear secretion test was performed by dropping a drop of 0.5% Alcaine anesthetics and then placing a standard filter paper (Sino-strips, Chauvin Pharmaceuticals Ltd., Kingston-Upon-Thames, England) in the conjunctiva sac. The tear-wetted filter paper was read 5 minutes later, and tear secretion dysfunction was diagnosed if the paper's wetting length was equal to or less than 5 mm. Both eyes of each participant were examined, and we defined a participant as having tear secretion dysfunction as long as one of the eyes showed abnormal test results. This study was carried out in compliance with the Helsinki Declaration, and verbal consent was obtained from all the participants. The study proposal was approved by the Research Committee of the Chi-Mei Medical Center.

We evaluated differences in the prevalence of tear secretion dysfunction using the chi-square or Fisher's exact test. Potential predictors of tear secretion dysfunction were evaluated by uni-variate and then multi-variate logistic regressions. In addition, some of the participants also received the same tear secretion test in the previous annual health examination, and we compare the results between these two tests by the McNemar's test. The statistical analyses were conducted using SPSS 12.0 software package, and all statistical tests were performed at the two-tailed significant level of 0.05.

## Results

During the study period, a total of 371 light-on test workers received the health examination at the clinic of the park, and 52 of them were excluded due to having ophthalmic diseases and other systemic diseases that may affect ophthalmic function. All the remaining 319 light-on test workers agreed to participate in the study. All the participants were women working by 4-shift rotations, and the average age was 24.2 years old (standard deviation [SD] = 3.8 years). They had a mean employment duration of 13.6 months (SD = 5.7 months), and 74 (23.2%) of them wore contact lens at work on a regular basis. Workers who wore contact lens at work had a higher prevalence of tear secretion dysfunction, but the differences in the prevalence were not statistically significant among the three age groups, between those who had engaged in light-on tests for more than one year and those who had not, or between the LCD and LCM station workers (Table 1).

Among the 11 ophthalmic symptoms evaluated, eye dryness was the most prevalent (prevalence = 43.3%) (Table 2). The prevalence was 32% for eye itching, 16% for red eyes, 11% for eye pain, 10.3% for blurred vision at far distance, 8.8% for increased discharge, 5.6% for floaters, 3.1% for double vision, 2.5% for unable to focus from near to far, 1.6% for change of color perception, and 1.6% for blurred vision at near distance. As many as 221 (69.3%) of the participants had more than one symptom. Participants with tear secretion dysfunction had a higher prevalence of eye dryness than those without the dysfunction (46.9% vs. 40.8%), but the increase was not statistically significant (p = 0.286).

The results of Schirmer's tests revealed that the overall prevalence of tear secretion dysfunction in at least one eye was 40.1% (128 cases). In the uni-variate analyses, only the use of contact lens appeared to be a significant predictor of tear secretion dysfunction, with an odds ratio (OR) of 1.69 (95% confidence interval [CI] = 1.00–2.86)(Table 3), but the prevalence of eye dryness among contact lens users were not significantly higher. After adjusting for age, workstation, and employment duration, we found the use of contact lens was still a significant predictor of tear secretion dysfunction, and the adjusted odds ratio (AOR) was 1.73 (95% CI = 1.02–2.94).

Among the 319 participants, 232 also received a Schirmer's test in the previous annual health examination in the same clinic using the same method. We compared their results of Schirmer's tests between the two years and found that 40 of the 156 participants (17.2%) with normal test results in 2000 turned abnormal in 2001. In contrast, only 21 of the 76 participants (9.1%) with abnormal test results in 2000 turned normal in 2001, and the difference was statistically significant (p = 0.02 for McNemar's test) (Table 4).

## Discussion

A previous study has shown that many clean room workers of semiconductor companies had eye strain, especially those who engaged in inspections using human eyes [6]. Our study also found a high prevalence of eye discomfort among woman workers engaged in light-on tests in clean rooms, and the most common symptom was eye dryness (prevalence = 43.3%), followed by eye itching (32.0%), eye pain (11.0%), and blurred vision (10.3%). These symptoms were similar to those observed in workers working with video display terminal (VDT). A study on long term VDT workers in Taiwan showed that the major eye symptoms were eye strain (82.4%), eye dryness (52.9%), eye itching (52.9%), and blurred vision (50.0%) [7]. Another study on VDT workers in Taiwan found that the most prevalent eye symptom was eye dryness (52.8%), followed by visual blurring (35.3%) and eye pain (33.9%) [8]. Light-on test workers are similar to VDT workers in that they need to look at screens during their work, and therefore it is not surprising that the two groups of workers share similar eye symptoms. However, whereas light-on test workers work in clean rooms with low illumination, VDT workers do not necessarily work in such environments. Furthermore, during the light-on test, the examiner needs to turn the screen to the brightest status, and therefore there are still some differences between these two groups of workers. In addition, all the light-on test workers in our study worked for 12 hours in each shift, which might also contribute to the development of the eye symptoms. A study in Taiwan suggested that dry eye symptoms in VDT workers might be contributable to the need to look at the screen and the low frequency of eye blinking [8]. A study on Japanese VDT workers found an association between eyes' blinking frequency and tear secretion, and both were lower among VDT workers in comparison with non-VDT workers [9]. Since the light-on test requires binocular gaze on the LCD panel and insufficient blinking frequency is common among workers using VDT, it is not surprising that eye dryness became the most common eye symptom in our study. A survey on eye dryness in 3722 Americans between 48 to 91 years of age (mean = 65 years old) found a prevalence of 14.4% [10], and a study of 2127 Japanese who sought for ophthalmology outpatient department service for the first time found 17% of the patients suffered from dry eye symptoms [11]. In contrast, the overall prevalence was up to 43.3% in our study.

In our study, participants with tear secretion dysfunction had a higher prevalence of eye dryness than those without the dysfunction (46.9% vs. 40.8), but the increase was not statistically significant (p = 0.286). This might indicate that the dysfunction was generally not severe enough to introduce a significant impact on the subjective symptoms. However, the mean employment duration of these workers was only 13.6 months, and therefore the results can only reflect relatively short term effects. In fact, a study also observed dry eye symptoms in VDT workers with enough tear secretion, because the lack of eye blinking may cause uneven tear distribution on the surface of eyeballs, which also contributes to dry eyes [12]. The finding is in line with our observation, and further follow-up of this population should be conducted to evaluate the long-term effects.

A study in Australia on 1584 participants showed a higher prevalence of dry eye symptoms in those who were over 40 years of age in comparison with those who were less than 40 years old (18.1% vs. 7.3%) [13]. Another study indicated that the amount of tear secretion decreased with age [14]. In our study, however, age did not appear to be a significant predictor of tear secretion dysfunction. This was probably due to the facts that our study participants were young (mean age 24.2 years old) and that some other risk factors of tear secretion dysfunction were prevalent in the population, which could be inferred by the high prevalence of tear secretion dysfunction (40.9%).

The logistic regression analyses in our study showed that the use of contact lens was an independent predictor of tear secretion dysfunction. A study of 2127 Japanese who sought for ophthalmology outpatient department service for the first time also found the prevalence dry eye symptoms was higher among contact lens users (p < 0.02) [12], which support our finding.

Whereas the employment duration did not appear to be associated the development of tear secretion dysfunction in the analyses of the 2001 test results alone (Table 3), the observation of the same group of participants over time demonstrated an increase in the prevalence of tear secretion dysfunction (Table 4). Because the second part of analyses took into account variations among individuals by using the same participant as her own control, the results are more reliable than simply comparing among a group of people at a particular time, as in the first part of analyses.

## Conclusion

The TFT-LCD industry has become a major industry in Taiwan and employed a large number of workers, many of whom, mostly women, are engaged in light-on tests. In this study, although the excess risk of tear secretion dysfunction in LCD workers in comparison with LCM workers, who had higher illumination in the work environment, did not reach statistical significance, we observed high prevalence of tear secretion dysfunction in both groups of workers, and in both groups the illumination was far under the lower limit of the range (200 to 500 Lux) generally recommended [15]. The low humidity in clean rooms [16], flashing light, low illumination of the work environment, and heavy use of the eye sight for long working hours might be factors of the high prevalence of tear secretion dysfunction and eye symptoms in this study population. In addition, we found the use of contact lens is an independent predictor of tear secretion dysfunction. Therefore, proper prevention measures should be taken to protect the eyes of these workers, including pre-employment training and adequate resting time. In addition, because the use of contact lens is a significant risk factor of tear secretion dysfunction and the environment and task can not be improved sufficiently to prevent the problem at the present time, these workers should be advised to avoid wearing contact lens during working hours. Intervention studies should be conducted to identify the effective prevention measures, and further follow-up should be conducted to evaluate the long-term health effects of the light-on test.

## Competing interests

The author(s) declare that they have no competing interests.

## Authors' contributions

SBS conceived and designed the study and was in charge of the recruitment of study participants. CWL helped designed the study and collected information on the work environment. JWS participated in the design of the study and performed the statistical analyses. SCK participated in the design of the study, helped examining the participants, and supervised the tear secretion tests. HRG participated in the design, supervised the conduct of the study and helped to draft the manuscript. All authors read and approved the final manuscript.

## Pre-publication history

The pre-publication history for this paper can be accessed here:



## Figures and Tables

**Table 1:** Prevalence of tear secretion dysfunction by demographic characteristics, 2000

Characteristics	Workers	Cases (%)	*p*
Age (year)			0.289
≤20	40	12 (30.0)	
21–25	171	74 (43.3)	
> 25	108	42 (38.9)	
Employment duration			0.610
≤1 year	144	60 (41.7)	
> 1 year	175	68 (38.9)	
Work station			0.452
LCD	102	44 (43.1)	
LCM	217	84 (38.7)	
Contact lens use			0.048
Yes	74	37 (50.0)	
No	245	91 (37.1)	
Total	319	128 (40.1)	

**Table 2:** The prevalence of ophthalmic symptoms by status of tear secretion function

Symptoms	Tear Secretion Function				*p*
		
	Normal (N = 191)		Dysfunction^a ^(N = 128)		
		
	Cases	%	Cases	%	
Eye dryness	78	40.8	60	46.9	0.286
Eye itching	64	33.5	38	29.7	0.473
Red eyes	28	14.7	23	18.0	0.429
Eye pain	22	11.5	13	10.2	0.703
Blurred vision at far distance	20	10.5	13	10.2	0.928
Increase discharge	18	9.4	10	7.8	0.618
Floaters	11	5.8	7	5.5	0.912
Double vision	5	2.6	5	3.9	0.517
Unable to focus from near to far	6	3.1	2	1.6	0.377
Change of color perception	0	0	0	0	
Blurred vision at near distance	4	2.1	1	0.8	0.355

^a^defined as having an abnormal Schirmer's test

**Table 3:** Factors affecting the tear secretion function

Variables	OR^a ^[95%CI^b^]	AOR^c ^[95%CI]
Age (year)	1.02 [0.96–1.08]	1.02 [0.96–1.08]
Employment (month)	0.99 [0.95–1.03]	0.99 [0.95–1.03]
Work at LCD stations	1.20 [0.75–1.94]	1.25 [0.77–2.03]
Contact lens use	1.69 [1.00–2.86]*	1.73 [1.02–2.94]*

^a^odds ratio

^b^confidence interval

^c^adjusted odds ratio; variables included in the model: age, employment, work at LCD stations, and contact lens use

*p < 0.05

**Table 4:** Comparison of Schirmer's test results of light-on test workers between 2000 and 2001

		Tear secretion test (2001) N (%)		
		
		Normal	Abnormal	Total
Tear secretion test (2000) N (%)	Normal	116 (50.0)	40 (17.2)	156 (67.2)
	Abnormal	21 (9.1)	55 (23.7)	76 (32.8)
	Total	137 (59.1)	95 (40.9)	232 (100.0)

McNemar's test *p *= 0.02
